# Developing positive behavioral skills among adolescents participating in basketball sports schools

**DOI:** 10.3389/fpsyg.2025.1444333

**Published:** 2025-06-10

**Authors:** Eimantas Pocius, Romualdas Malinauskas

**Affiliations:** Department of Physical and Social Education, Lithuanian Sports University, Kaunas, Lithuania

**Keywords:** adolescents, basketball sports schools, positive emotional skills, positive personal skills, positive social skills, skills training program

## Abstract

**Introduction:**

The aim of this study is to evaluate the impact of a positive behavior skills development program on adolescents who participate in basketball sports schools.

**Methods:**

Sixty-two adolescent athletes (Mage 15.83 ± 0.37) took part in this educational experiment. Participants were randomly selected from 2 basketball sports schools chosen from a list of basketball sports schools and divided into experimental (*n* = 30) and control (*n* = 32) groups. The experimental group participants were subjected to the effect of the positive behavior skills development program, which aimed to develop positive behavior skills among adolescents participating in basketball sports schools, including taking responsibility, positive self-evaluation, prosocial behavior with teammates, prosocial behavior with opponents, taking social responsibility, cooperation, assertiveness, empathy, self-control, ability to evaluate and convey emotions, ability to utilize one’s positive emotional experience, ability to comprehend and analyze emotions, and ability to control emotions. Positive behavior skills development activities were conducted by one of the researchers together with the sports school coaches. At the beginning and end of the educational experiment, participants from both the experimental and control groups completed the following questionnaires: Personal and Social Responsibility Questionnaire, Rosenberg Self-Esteem Scale, The Prosocial and Antisocial Behavior in Sport Scale, Social Skills Rating System-Secondary Student form, and Schutte Self-Report Inventory.

**Results:**

The positive behavior skills development activities implemented during the educational experiment had a positive and statistically significant impact on the positive behavior skills of the experimental group adolescents.

**Discussion:**

The findings of the present study could be useful for educational programs developers who may consider integrating elements of this educational program into wider education systems.

## 1 Introduction

Positive behavior skills are defined as skills that allow individuals to create personal well-being by interacting with others or groups of people, adapting to the requirements of different environments or cultures (Pearson et al., 2021). Positive behavior skills are part of the paradigm of positive youth development, which is a conceptual approach to developmental research and practice emphasizing the cultivation of adolescent strengths and potential (Holt et al., 2020; Lerner, 2017). The results of scientific studies show that the development of positive behavior skills can prevent negative behaviors in adolescence and thus contribute to positive adolescent development (Qi et al., 2020). It has been found that through the development of positive behavior skills, individuals’ self-regulation increases, interpersonal skills strengthen, problematic and aggressive behavior decreases, and as a result, relationships with peers and adults improve (Catalano et al., 2002; Strachan et al., 2011). This is particularly important because adolescence exhibits perhaps the greatest expression of negative behavior compared to other stages of human life. Especially around the ages of 15–16, adolescents exhibit typical behavior related to seeking various sensations and a desire to experience new and risky experiences (e.g., using psychoactive substances, engaging in sexual behavior) (Ball et al., 2023). Unfortunately, half of the criminals commit their first offense between the ages of 14 and 17 (Ball et al., 2023; Bergin et al., 2018). The development of positive behavior skills is recognized as a crucial factor through which adolescents become productive members of society (Camire and Santos, 2019). The development of positive behavior skills focuses on the interaction between individuals and specific environments, such as teams, families, and schools. This interaction helps individuals apply their experiences across different settings (Qi et al., 2020). During the development of positive behavior skills, self-control, social skills (Hermens et al., 2017), teamwork (Lower-Hoppe et al., 2020), communication (Holt et al., 2017), personal and social responsibility skills are developed (Whitley et al., 2019). Sport is considered the primary activity for developing positive behavior skills (Bruner et al., 2022; Johnston et al., 2013; Larson et al., 2006). The success of skills development through sports depends on the time dedicated to the activity, the structure and rules of the sport, and whether the adolescent participates voluntarily rather than under coercion (Jones and Parker, 2013). Strachan et al. (2011) identify seven key factors that facilitate the opportunities for the development of positive behavior skills: (1) physical and psychological safety, (2) structured activity, (3) supportive relationships, (4) opportunities to belong to a group, (5) positive social norms, (6) support for efficacy and significance, (7) opportunities to develop skills that can be applied in other environments such as family, school, and community. It is evident that sports are an environment characterized by these factors: (1) sports allow individuals to feel physically and psychologically secure due to interaction with peers, (2) sports involve structured activities (e.g., sports programs) and discipline (e.g., rules), (3) there is support from coaches or team members, (4) individuals belong to various groups in sports (e.g., team, representatives of sports branches, national teams), (5) sports promote positive social norms (e.g., fairness, respect, values), (6) sports aim for results, so the activity must be effective and constantly meaningful (for the athlete, coach, and family members), (7) various skills can be developed during sports practices and competitions, which can be applied in other environments (e.g., collaboration, emotional control, perseverance). Various sports activities are oriented towards collaboration and working together towards a goal, thus sports provide an excellent environment for developing skills related to teamwork and decision-making (Perkins and Noam, 2007; Waid and Uhrich, 2020). Sports can create conditions for developing skills that are oriented towards interpersonal relationships, thus helping adolescents integrate into adult social networks (Hansen et al., 2010). According to Strachan et al. (2011), sports provide an excellent environment for studying positive behavior skills among young athletes. One of the sports branches where these factors can be implemented is basketball, which is the dominant and most popular sport among adolescents (especially boys) in Lithuania. Research data shows that risky and socially unacceptable behavior is more common among male adolescents (Lipowski et al., 2016; Schuster et al., 2013; Veselska et al., 2009; Zuckerman et al., 2021). Therefore, summarizing various scientific literature data, it can be assumed that positive behavior skills should be fostered in basketball sports schools. Given that ages 14–18 show the highest expression of negative behaviors, adolescents aged 13–18 are less focused on forming new attachments, and popular extracurricular activities like sports become important (Bergin et al., 2018), along with the peak in information processing speed occurring between ages 15–18 (Coyle et al., 2011), it is reasonable to assume that positive behavior skills should be fostered in 15-16-year-old adolescents in basketball sports schools. The aim of this study is to evaluate the impact of a positive behavior skills development program on adolescents who participate in basketball sports schools.

Opstoel et al. (2020), after conducting a comprehensive analysis of scientific literature, assert that various skills acquired through sports have a positive impact on personal and social development. However, most skills development programs are oriented solely towards physical education classes, with the greatest emphasis placed on fostering prosocial behavior and cooperation skills, rather than other important skills. In this study, we aim to address several existing gaps in the literature. Firstly, in Lithuania, adolescents tend to participate more frequently in various extracurricular sports activities (sports schools, sports clubs) while often skipping physical education classes. Consequently, there are barriers to fostering positive behavior skills among adolescents through physical education classes. Therefore, in this study, we developed a positive behavior skills development program tailored to adolescents who participate in basketball sports schools. Typically, youth development research emphasizes life skills that can be readily applied in various life contexts. In the scientific literature on positive behavior skills development (Hemphill et al., 2019; Holt et al., 2020; Napolitano et al., 2021; Soto et al., 2021; Filiz and Demirhan, 2019; Midura and Glover, 2005; Watson and Clocksin, 2013), it becomes evident that positive behavior skills encompass personal, social, and emotional skills that are crucial for achieving the goals of the positive youth development paradigm. Thus, our developed youth development program encompasses the main groups of positive behavior skills –– positive personal skills, positive social skills, and positive emotional skills. In doing so, we aim to address the scientific gap related to the limited skill development often encountered in skills development programs aimed at implementing the conceptual principles of positive youth development, which emphasize the “plasticity” of human development. At the same time, we recognize that all young people have the potential to change their behavior, and that this behavior change is not only crucial for preventing unwanted/problematic behavior (Armour and Sandford, 2013), but also essential for achieving socially desirable positive behavior (Majed et al., 2022). Based on the above-mentioned studies, we presume that a comprehensive program of positive behavioral skills development for adolescents playing basketball in schools would be effective if it included the development of the following skills: positive personal, positive social and positive emotional.

## 2 Methods

### 2.1 Context and participants

The sample size was calculated by the G*Power program (Kang, 2021), taking into account an alpha error rate of 5% and a beta error rate of 20%. The participants of the educational experiment were selected through probability sampling. A three-stage cluster selection was conducted (Cohen et al., 2018; Hutchison and Styles, 2010). Firstly, an alphabetical list of basketball sports schools in Lithuania was compiled. Then, a table of numbers was created, which was filled with school numbers obtained through a coded list of sports schools. Based on this table, selected sports schools were contacted, and all male adolescents (aged 15–16) from these schools were included in the study. The sample for educational experiment did not include both males and females, because men’s basketball is the dominant sport in the country and women’s basketball has only a few sports schools. It was decided to select only men’s basketball sports schools in order to avoid probability selection biases. Inclusion criteria required male students (aged 15–16) who are regular participants in a sports school and have given their consent to participate in the study, while exclusion criteria included those who discontinued attending training sessions**.”**

One sports school declined to participate in the educational experiment. In total, 63 adolescent athletes from 2 sports schools were included in the educational experiment. Then, all adolescent athletes were assigned to one of four groups through a random selection process. Finally, through random selection from those four groups, two were assigned to the experimental group (*n* = 31) and two to the control group (*n* = 32). During the educational experiment, one participant from the experimental group discontinued attending training sessions, resulting in a total of 62 adolescent athletes participating in the educational experiment: 30 in the experimental group and 32 in the control group. The mean age of the total sample of adolescents at the beginning of the study was 15.83 ± 0.37 years. The ages of participants in the experimental and control groups at the beginning of the study were homogeneous: 15.87 ± 0.35 years for the experimental group and 15.81 ± 0.40 years for the control group.

### 2.2 Instruments

During the study, reliable and locally adapted questionnaires were selected to assess the skill sets relevant to the study context or directly related (specifically developed tools) to the sets of positive behavior skills under investigation.

To assess Taking responsibility and Taking social responsibility skills, the *Personal and Social Responsibility Questionnaire (PSRQ)* by Li et al. (2008) was chosen. This questionnaire is a reliable tool for determining personal and social responsibility in a sports context and is recommended for use in research within the positive youth development paradigm, aimed at creating or improving skills development programs that aim to cultivate personal and social responsibility skills (Li et al., 2008). The questionnaire consists of 14 statements, with 7 statements assigned to each of the two questionnaire scales. The Taking responsibility scale comprises statements 1 to 7, while the Taking social responsibility scale comprises statements 8 to 14, with statement 14 being reverse-scored. All questionnaire statements are rated on a 6-point Likert scale: 1 – strongly disagree, 2 – disagree, 3 – slightly disagree, 4 – slightly agree, 5 – agree, 6 – strongly agree (Li et al., 2008; Martins et al., 2015). The Personal and Social Responsibility Questionnaire has been adapted in Lithuania, with a Cronbach’s alpha coefficient value of 0.81 (Juodsnukis and Malinauskas, 2014).

For assessing Positive self-evaluation skills, the *Rosenberg Self-Esteem Scale (RSES)* by Ciarrochi and Bilich (2006) and Rosenberg (1965) was chosen. This questionnaire is the most widely used tool worldwide for assessing individuals’ positive self-esteem (Garcia et al., 2019; Jordan, 2020). The tool is used in research on positive behavior skills development among adolescents (Mohammadzadeh et al., 2017; Seema and Kumar, 2018) as well as in various sports-related studies (Dahiya and Gupta, 2021; Koszałka-Silska et al., 2021a; Šagat et al., 2021). The Rosenberg Self-Esteem Scale consists of 10 statements. The statements are rated on a 4-point Likert scale as follows: for statements 1, 2, 4, 6, and 7, 3 – strongly agree, 2 – agree, 1 – disagree, 0 – strongly disagree, while for statements 3, 5, 8, 9, and 10, the scoring is reversed (Ciarrochi and Bilich, 2006; Rosenberg, 1965). The Rosenberg Self-Esteem Scale has been adapted in Lithuania, with a Cronbach’s alpha coefficient value of 0.73 (Juodsnukis and Malinauskas, 2014).

To assess skills of Prosocial behavior with teammates and Prosocial behavior with opponents, *The Prosocial and Antisocial Behavior in Sport Scale (PABSS)* by Kavussanu and Boardley (2009) was used. Unlike other prosocial and antisocial behavior questionnaires, this scale was exclusively developed for the sports context. The first scale, antisocial behavior with opponents, consists of statements 13, 14, 15, 16, 17, 18, 19, and 20. The second scale comprises statements 8, 9, 10, 11, and 12, revealing antisocial behavior with the team. The third scale, Prosocial behavior with teammates, consists of statements 1, 2, 3, and 4. The fourth scale, Prosocial behavior with opponents, is associated with statements 5, 6, and 7. Each scale’s statements are summed and divided by the total number of statements. Each questionnaire statement is rated on a 5-point Likert scale: 1 – never; 2 – rarely; 3 – sometimes; 4 – often; 5 – very often (Kavussanu and Boardley, 2009; Kavussanu et al., 2013). The Prosocial and Antisocial Behavior in Sport Scale has been adapted in Lithuania, with Cronbach’s alpha coefficient values ranging from 0.79 to 0.85 (Šukys, 2010). Since this study focused on Prosocial behavior with teammates and Prosocial behavior with opponents, the corresponding scales from the PABSS questionnaire were utilized.

To assess cooperation, assertiveness, empathy, and self-control skills, the *Social Skills Rating System-Secondary Student form (SSRS-S)* by Gresham and Elliott (1990) was chosen. This questionnaire was selected for this study because the construct of positive social skills in this study was based on the authors’ conception of social skills (Gresham et al., 2011). Thus, the Social Skills Rating System-Secondary Student form is the most suitable and reliable instrument for assessing the positive social skills under investigation in this study. The questionnaire consists of 39 statements, with a certain number assigned to four scales: (1) cooperation scale includes statements 6, 9, 13, 14, 17, 20, 31, 35, 36, and 37; (2) assertiveness scale includes statements 1, 3, 4, 16, 23, 26, 30, 33, and 38; (3) empathy scale includes statements 2, 5, 8, 12, 21, 24, 25, 28, 29, and 39; and (4) self-control scale includes statements 7, 10, 11, 15, 18, 19, 22, 27, 32, and 34. Each questionnaire statement is rated on a 3-point Likert scale: 0 – never, 1 – sometimes, 2 – very often (Elliott et al., 2015; Gresham and Elliott, 1990). The Social Skills Rating System-Secondary Student form has been adapted in Lithuania, with a Cronbach’s alpha coefficient value of 0.70 (Akelaitis, 2015).

For assessing positive emotional skills (ability to evaluate and convey emotions, ability to utilize one’s positive emotional experience, ability to comprehend and analyze emotions, ability to control emotions), the *Schutte Self-Report Inventory (SSRI)* by Schutte et al. (1998) was selected. Through this questionnaire, emotional intelligence is measured as positive emotional skills (Palmer, 2003). This tool was developed based on the emotional intelligence model by Salovey and Mayer (1990), which forms the construct of positive emotional skills in this study. Additionally, the reliability and validity of this tool have been demonstrated in the field of sports research (Lane et al., 2009). The questionnaire consists of 33 statements and has four scales corresponding to emotional skills. Statements assigned to each scale are as follows: ability to evaluate and convey emotions scale includes statements 1, 5, 15, 18, 25, 29, 32, and 33 (with statements 5 and 33 scored in reverse), ability to utilize one’s positive emotional experience scale includes statements 2, 3, 4, 7, 10, 14, 16, 17, 20, 23, 24, 27, 28, and 31 (with statement 28 scored in reverse), ability to comprehend and analyze emotions scale includes statements 9, 10, 12, 19, 21, and 22, and ability to control emotions scale includes statements 11, 13, 26, 27, and 30. Two questionnaire statements are assigned to two scales: statement 10 to both ability to utilize one’s positive emotional experience and ability to comprehend and analyze emotions scales, while statement 27 to both ability to utilize one’s positive emotional experience and ability to control emotions scales. Each questionnaire statement is rated on a 5-point Likert scale: 1 – strongly disagree, 2 – disagree, 3 – neither agree nor disagree, 4 – agree, 5 – strongly agree (Palmer, 2003; Schutte et al., 1998). The Schutte Self-Report Inventory has been adapted in Lithuania, with a Cronbach’s alpha coefficient value of 0.76 (Akelaitis and Malinauskas, 2014).

### 2.3 Intervention

Experimental pretest-posttest design was chosen for the study. To evaluate the effectiveness of a positive behavior skills training program designed for adolescents who participate in basketball sports schools, we conducted an educational experiment. During the educational experiment, participants in the experimental research group were subjected to skills training activities, while participants in the control group did not receive any social skills activities. The positive behavior skills development program consisted of 13 activities. Each activity was aimed at the development of a specific skill (except for the prosocial behavior skill, which was additionally divided into two additional skills: prosocial behavior with teammates and prosocial behavior with opponents). The duration of each activity was 60 min, so in total, positive behavior skills for adolescents were trained for 13 h. The frequency of activities was once a week. Positive behavior skills for adolescents in the control group were trained before basketball practice. Positive behavior skills for adolescent athletes were trained in stages. First, the skill description stage was implemented, during which new sports exercises or special tasks were provided, and the young athletes were introduced to the effects of the exercise/task explanation. Then, at the skill demonstration stage, the skill was demonstrated practically to the young athletes, i.e., explained in practice. In the skill practice stage at basketball sports schools, adolescent athletes themselves tried to perform exercises correctly so that they could use them in other situations. Finally, in the skill consolidation stage, adolescents were encouraged to apply the acquired skill in another natural environment, for example, at home or at school, by giving them certain tasks (e.g., homework) (Bean et al., 2018; Bierman et al., 2017; Huysmans et al., 2021; Newman et al., 2021; Pierce et al., 2017). The first table provides more information about the skills comprising the training program (the structure of the training program), the goals of skill training, and the training methods. For example, when training the “taking social responsibility” skill for adolescents, the skill was explained and described. Then, adolescents were involved in a discussion during which a discussion question (situation) was asked, and they were also asked to provide feedback on how they imagine what the “taking social responsibility” skill is. After clarifying and discussing what the “taking social responsibility” skill entails, adolescents performed a skill training activity. Adolescent athletes divided into smaller groups and performed activity. During activity execution, they blindfolded one team member and instructed them to transport the ball from one side of the court to the other through various obstacles. Each group member could give one command at a time so that the common goal could be achieved – i.e., the group member blindfolded would complete the task as quickly as possible. After completing the activity, adolescents were encouraged to provide feedback on the experiences they had during the activity. Finally, the adolescent athletes were assigned homework. The homework assignment required adolescents to create a family household chores list at home, which each member should responsibly carry out for the common well-being of the family. Meanwhile, in training the “Ability to utilize one’s positive emotional experience” skill, methods such as the breathing method and imagery method were employed. To enable adolescents to utilize their previously experienced positive emotions, they performed breathing exercises, which allowed them to relax more deeply. Deep breathing exercises also helped them concentrate better during visualization. During visualization exercises, adolescent athletes closed their eyes and had to recall basketball games they lost, as well as negative emotions they experienced at that time. Remembering these emotions, they were encouraged to recall moments when they felt positive. Recalling these mental images, adolescents were encouraged to reinforce them in their minds by repeating various affirmations such as “*I remember a time when I was in a good mood and calm*,” “*I remember a time when I was happy*,” “*I remember a time when I was relaxed*,” “*I am in a good mood, calm, and relaxed*.” The activities of the training program were aimed at revealing adolescents’ potential to behave positively by emphasizing their strengths, and during skills training sessions, activities were aimed not only at training specific skills but also at reflecting on the importance of these skills in other life contexts and their application in them. This way, we implemented the conceptual attitudes of positive youth development paradigms (Holt et al., 2020; Lerner, 2017).

### 2.4 Data collection

Before conducting the study, necessary procedures were performed. The study adhered to the principles outlined in the Helsinki Declaration and obtained approval from the Ethics Committee of the Lithuanian Sports University, with approval number SMTEK-47, granted on June 3, 2021. The study was conducted in stages. Initially, a preliminary study was conducted, during which the experimental and control group participants completed skill assessment questionnaires provided for the study. Then, the experimental group participants underwent the intervention (development program). Finally, at the end of the intervention, a repeated assessment of both experimental and control group participants was conducted using the same questionnaires employed at the beginning of the study (the same questionnaires). The questionnaires were anonymous and only included general information about the participants (age) and their consent to participate in the study. Participants were given as much time as they wished to complete the questionnaires. On average, participants took between 20 to 30 min to complete the questionnaires.

### 2.5 Data analysis

Statistical analyses were conducted utilizing SPSS software (version 29.0). The study’s experimental setup involved quantitatively analyzing data from both the experimental and control groups. Mean (*M*) and standard deviation (*SD*) computations were conducted for each variable. Skewness and kurtosis coefficients were used to assess data normality via multivariate analysis of variance (MANOVA), with skewness and kurtosis values typically falling between −2 and 2 for normal data. Pearson’s correlations (two-tailed) were computed for all variables. The independent samples *t-*test was employed to compare the means of the experimental and control groups to determine if there was a statistically significant difference between them. A repeated measures (RM) MANOVA was utilized, employing a 2 (Group: experimental and control) × 2 (Time: pre- and post-experiment) design, followed by one-way ANOVA, to investigate Group and Time interactions concerning positive behavioral skills. It must be noted that a more powerful ANCOVA procedure could not be applied in this study because it was determined using the G*Pover software (Kang, 2021), that if 52 participants are enough for RM MANOVA (the total sample size in the study is 62 participants, which is entirely sufficient), then at least 269 study participants would be required for ANCOVA. Preliminary examinations for multicollinearity, sphericity, homogeneity, and equality of variance/covariance matrices revealed no significant concerns. Multivariate effects were assessed using Wilks’ Lambda statistics, with a significance threshold of 0.05. The effect sizes for F-statistics were reported as partial eta-squared (*η*_p_^2^) (Table 1).

**Table 1:** Description of a development program for positive behavioral skills for adolescents participating in basketball sports schools.

Intervention	Number of sessions	Skill	Goals	Training methods
Positive personal skills	1	Taking responsibility	Learn to take responsibility for your own level of physical activity and the overall well-being of the team.	Explanation, description, discussion, giving feedback, method of active games, small group method, completing homework.
	1	Positive self-evaluation	Learn how to analyze and assess a person’s strengths or areas for improvement.	Explanation, description, discussion, giving feedback, case study, completing homework.
	2	Prosocial behavior	Learn how to encourage teammates, congratulate teammates for a good game, give positive feedback to teammates, give constructive feedback to teammates, ask to stop the game/match when an opponent is injured and help an opponent get up from the floor.	Explanation, description, discussion, giving feedback, case study, completing homework.
Positive social skills	1	Taking social responsibility	Learn to take shared (team) responsibility through the ability to share, the ability to help, the ability to complete tasks, the ability to follow instructions or directions.	Explanation, description, discussion, giving feedback, small group method, completing homework.
	1	Cooperation	Learn to work together with others towards a common goal.	Explanation, description, discussion, giving feedback, method of active games, small group method, completing homework.
	1	Assertiveness	Learn to persevere in the pursuit of a goal, even though the road to it is long and difficult, and even though it requires the exertion of every effort and the overcoming of many setbacks and obstacles.	Explanation, description, case study, giving feedback, completing homework.
	1	Empathy	Learn to understand another person’s feelings or emotions and to communicate this understanding, helping the other person to feel understood.	Explanation, description, giving feedback, role-play method, case study, completing homework.
	1	Self-control	Learn to monitor their health, physical development, physical fitness, mental state, emotions, behaviors and actions in a purposeful way, and to analyze and correct them in the process of physical education.	Explanation, description, giving feedback, case study, small group method, completing homework.
Positive emotional skills	1	Ability to evaluate and convey emotions	Learn to recognize your own emotions and those of others (e.g., teammates), recognize emotions in objects (e.g., pictures) and be able to express emotions accurately.	Explanation, description, giving feedback, role-play method, case study, completing homework.
	1	Ability to utilize one’s positive emotional experience	Learn how to use emotions in a way that prioritizes thoughts and uses emotions as a tool to help solve problems.	Explanation, description, giving feedback, discussion, breathing method, imagery method, completing homework.
	1	Ability to comprehend and analyze emotions	Learn to accurately attribute emotions, understand emotions and their relationships, and understand complex feelings and changes in emotions.	Explanation, description, giving feedback, breathing method, mindfulness method, completing homework.
	1	Ability to control emotions	Learn how to successfully manage your own and others’ emotions.	Explanation, description, giving feedback, discussion, breathing method, imagery method, completing homework.

## 3 Results

To assess the impact of the educational program on the development of positive behavior skills among adolescents participating in basketball sports schools, we first conducted a descriptive statistical analysis of the overall study sample (Table 2). We calculated the mean, standard deviation, skewness, skewness standard error, kurtosis, and kurtosis standard error of the sample’s results before and after the intervention. The obtained data confirmed a normal distribution of the data both before and after the intervention, as all skewness values (ranging from −0.63 to 0.45) and kurtosis values (ranging from −0.94 to 1.62) fell within the range of −2 to +2 (Garson, 2012; George and Mallery, 2010).

**Table 2:** Means, standard deviations, and normality tests of the study variables (*N* = 62).

Positive behavioral skills	Before experiment						After experiment					
	*M*	*SD*	Sk	SkSE	Ku	KuSE	*M*	*SD*	Sk	SkSE	Ku	KuSE
Taking responsibility	30.26	3.70	−0.03	0.30	−0.48	0.60	32.10	3.92	−0.16	0.30	0.75	0.60
Positive self-evaluation	16.80	2.15	−0.63	0.30	1.62	0.60	15.98	1.83	−0.23	0.30	−0.51	0.60
Prosocial behavior with teammates	4.08	0.49	0.14	0.30	−0.16	0.60	4.19	0.55	−0.17	0.30	−0.14	0.60
Prosocial behavior with opponents	2.83	0.72	0.27	0.30	0.25	0.60	3.15	0.80	−0.27	0.30	−0.39	0.60
Taking social responsibility	28.87	4.17	0.45	0.30	−0.47	0.60	30.74	4.42	−0.04	0.30	0.23	0.60
Cooperation	13.05	2.81	0.04	0.30	−0.74	0.60	14.27	2.96	0.07	0.30	−0.65	0.60
Assertiveness	11.40	2.95	−0.24	0.30	−0.47	0.60	12.53	2.55	−0.16	0.30	−0.31	0.60
Empathy	14.47	2.96	0.15	0.30	−0.94	0.60	15.74	2.93	−0.42	0.30	−0.76	0.60
Self-control	13.19	2.54	−0.02	0.30	−0.83	0.60	14.34	2.75	0.02	0.30	−0.75	0.60
Ability to evaluate and convey emotions	28.37	3.34	−0.04	0.30	0.59	0.60	29.27	3.13	−0.40	0.30	0.10	0.60
Ability to utilize one’s positive emotional experience	53.82	4.85	−0.03	0.30	−0.12	0.60	55.11	4.47	−0.23	0.30	0.19	0.60
Ability to comprehend and analyze emotions	21.95	2.25	−0.17	0.30	−0.007	0.60	22.60	2.36	0.02	0.30	−0.58	0.60
Ability to control emotions	16.05	2.36	0.07	0.30	0.22	0.60	16.70	2.37	0.11	0.30	0.18	0.60

Sk – Skewness; SkSE - Skewness standard error; Ku – Kurtosis; KuSE - Kurtosis standard error. M – Means; SD – standard deviations.

To establish the validity of the educational experiment, we compared the statistical indicators of positive behavior skills among adolescents in the experimental and control groups before and after the intervention. The application of the independent samples *t*-test revealed that prior to the educational experiment, the statistical indicators of positive behavior skills did not significantly differ between the experimental and control groups: taking responsibility (*t*_60_ = 0.02; *p* = 0.99), positive self-evaluation (*t*_60_ = −0.00; *p* = 0.99), prosocial behavior with teammates (*t*_60_ = 0.75; *p* = 0.46), prosocial behavior with opponents (*t*_60_ = −0.53; *p* = 0.60), taking social responsibility (*t*_60_ = −0.10; *p* = 0.92), cooperation (*t*_60_ = −0.04; *p* = 0.97), assertiveness (*t*_60_ = −0.01; *p* = 0.99), empathy (*t*_60_ =. 17; *p* = 0.87), self-control (*t*_60_ = −0.08; *p* = 0.94), ability to evaluate and convey emotions (*t*_60_ = 0.29; *p* = 0.77), ability to utilize one’s positive emotional experience (*t*_60_ = 0.75; *p* = 0.46), ability to comprehend and analyze emotions (*t*_60_ = 0.39; *p* = 0.70), ability to control emotions (*t*_60_ = 0.06; *p* = 0.95). So, the results confirm the validity of the intervention experiment.

We analyzed Pearson’s bivariate correlation coefficient to explore the relationships between variables. The correlation coefficients, illustrating these connections, are provided separately for the periods before and after the experiment in Table 3. This analysis aimed to investigate the associations between each dependent variable, taking into account the potential presence of multicollinearity. Importantly, none of the correlations exceeded 0.85, confirming the validity of assuming multicollinearity (Kline, 2005).

**Table 3:** Correlations of dependent variables.

Positive behavioral skills	1	2	3	4	5	6	7	8	9	10	11	12	13
Taking responsibility	1	0.422^**^	0.414^**^	0.409^**^	0.505^**^	0.517^**^	0.529^**^	0.551^**^	0.554^**^	0.231^**^	0.475^**^	0.373^**^	0.501^**^
Positive self-evaluation	0.422^**^	1	0.213^*^	0.255^**^	0.269^**^	0.491^**^	0.520^**^	0.478^**^	0.460^**^	0.256^**^	0.325^**^	0.284^**^	0.124
Prosocial behavior with teammates	0.414^**^	0.213^*^	1	0.096	0.376^**^	0.319^**^	0.315^**^	0.515^**^	0.351^**^	0.413^**^	0.424^**^	0.066	0.287^**^
Prosocial behavior with opponents	0.409^**^	0.255^**^	0.096	1	0.398^**^	0.261^**^	0.146	0.200^*^	0.129	0.124	0.221^*^	0.018	0.308^**^
Taking social responsibility	0.505^**^	0.269^**^	0.376^**^	0.398^**^	1	0.425^**^	0.493^**^	0.417^**^	0.325^**^	0.401^**^	0.490^**^	0.300^**^	0.502^**^
Cooperation	0.517^**^	0.491^**^	0.319^**^	0.261^**^	0.425^**^	1	0.618^**^	0.598^**^	0.703^**^	0.181^*^	0.303^**^	0.454^**^	0.381^**^
Assertiveness	0.529^**^	0.520^**^	0.315^**^	0.146	0.493^**^	0.618^**^	1	0.720^**^	0.519^**^	0.352^**^	0.455^**^	0.483^**^	0.474^**^
Empathy	0.551^**^	0.478^**^	0.515^**^	0.200^*^	0.417^**^	0.598^**^	0.720^**^	1	0.501^**^	0.282^**^	0.488^**^	0.413^**^	0.397^**^
Self-control	0.554^**^	0.460^**^	0.351^**^	0.129	0.325^**^	0.703^**^	0.519^**^	0.501^**^	1	0.265^**^	0.369^**^	0.490^**^	0.372^**^
Ability to evaluate and convey emotions	0.231^**^	0.256^**^	0.413^**^	0.124	0.401^**^	0.181^*^	0.352^**^	0.282^**^	0.265^**^	1	0.401^**^	0.290^**^	0.214^*^
Ability to utilize one’s positive emotional experience	0.475^**^	0.325^**^	0.424^**^	0.221^*^	0.490^**^	0.303^**^	0.455^**^	0.488^**^	0.369^**^	0.401^**^	1	0.490^**^	0.488^**^
Ability to comprehend and analyze emotions	0.373^**^	0.284^**^	0.066	0.018	0.300^**^	0.454^**^	0.483^**^	0.413^**^	0.490^**^	0.290^**^	0.490^**^	1	0.257^**^
Ability to control emotions	0.501^**^	0.124	0.287^**^	0.308^**^	0.502^**^	0.381^**^	0.474^**^	0.397^**^	0.372^**^	0.214^*^	0.488^**^	0.257^**^	1

Correlations below the diagonal are for Time before experiment. Correlations above the diagonal are for Time after experiment. *N* = 62. *p < 0.05; **p < 0.01.

To investigate the effects of the positive behavioral skills development program on adolescents participating in basketball sports schools, we conducted a repeated measures MANOVA with a 2 (Group) × 2 (Time) design. A crucial prerequisite for the univariate RM-MANOVA procedure is the assumption of sphericity. The requirement for Mauchly’s test of sphericity was satisfied since the RM variables have only two levels. The RM-MANOVA analysis demonstrated notable impacts of the positive behavioral skills development program on adolescents’ positive behavioral skills. Specifically, the Group by Time interaction exerted a significant influence (Wilks’ Lambda = 0.54; *F* (13,48) = 3.14; *p* < 0.01; *η*_p_^2^ = 0.46). From the statistical indicators presented in Table 4, it is possible to observe a statistically significant effect of the development program on the development of taking responsibility (*p* < 0.01), positive self-evaluation (*p* < 0.01), prosocial behavior with teammates (*p* < 0.05), prosocial behavior with opponents (*p* < 0.01), taking social responsibility (*p* < 0.01), cooperation (*p* < 0.01), assertiveness (*p* < 0.01), empathy (*p* < 0.01), self-control (*p* < 0.01), ability to evaluate and convey emotions (*p* < 0.05), ability to utilize one’s positive emotional experience (*p* < 0.05), ability to comprehend and analyze emotions (*p* < 0.05) and ability to control emotions (*p* < 0.05) in adolescents participating in sports schools.

**Table 4:** Indicators of positive behavioral skills of adolescents participating in basketball sports schools before and after the educational experiment.

Positive behavioral skills	Experimental group *M (SD)*		Control group *M (SD)*		Univariate tests of RM MANOVA Group × Time		
	Before experiment	After experiment	Before experiment	After experiment	*F*_1.60_	*p*	η_p_^2^
Taking responsibility	30.27 (3.69)	33.87 (3.41)	30.25 (3.78)	30.44 (3.66)	10.91**	0.002	0.154
Positive self-evaluation	15.967 (2.27)	17.63 (1.67)	15.969 (1.84)	15.94 (1.81)	12.25**	0.001	0.170
Prosocial behavior with teammates	4.13 (0.57)	4.43 (0.44)	4.03 (0.42)	3.97 (0.56)	5.27*	0.025	0.081
Prosocial behavior with opponents	2.78 (0.72)	3.38 (0.72)	2.88 (0.74)	2.94 (0.82)	7.28**	0.009	0.108
Taking social responsibility	28.87 (4.22)	32.63 (4.60)	28.97 (4.20)	28.97 (3.47)	11.56**	0.001	0.162
Cooperation	13.03 (3.30)	15.63 (2.63)	13.06 (2.33)	13.00 (2.70)	21.17**	0.000	0.261
Assertiveness	11.40 (3.13)	13.77 (2.22)	11.41 (2.83)	11.38 (2.30)	13.99**	0.000	0.189
Empathy	14.53 (2.85)	17.10 (1.86)	14.41 (3.11)	14.47 (3.20)	21.01**	0.000	0.259
Self-control	13.17 (2.96)	15.57 (2.24)	13.22 (2.12)	13.19 (2.72)	17.82**	0.000	0.229
Ability to evaluate and convey emotions	28.50 (2.99)	30.43 (2.46)	28.25 (3.68)	28.19 (3.33)	5.90*	0.018	0.089
Ability to utilize one’s positive emotional experience	54.30 (5.45)	57.10 (4.05)	53.38 (4.26)	53.25 (4.06)	5.82*	0.019	0.088
Ability to comprehend and analyze emotions	22.07 (2.21)	23.37 (2.44)	21.84 (2.32)	21.88 (2.08)	5.21*	0.026	0.080
Ability to control emotions	16.07 (1.66)	17.40 (2.39)	16.03 (2.89)	16.06 (2.20)	5.15*	0.027	0.079

η_p_^2^ – partial eta squared; * *p* < 0.05; ** *p* < 0.01.

## 4 Discussion

The results of the intervention experiment confirmed the hypothesis that the positive behavior skills development program designed for adolescents would be effective if it consisted of positive personal, positive social, and positive emotional skills, and if the program were implemented in basketball sports schools. After the implementation of the training program, the experimental group adolescents, who were involved in basketball sports schools, showed statistically significantly higher skill scores compared to the control group adolescents in all skill scales: taking responsibility (a medium effect: *η*_p_^2^ = 0.154), positive self-evaluation (a medium effect: *η*_p_^2^ = 0.170), prosocial behavior with teammates (a small effect: *η*_p_^2^ = 0.081), prosocial behavior with opponents (a small effect: *η*_p_^2^ = 0.108), taking social responsibility (a medium effect: *η*_p_^2^ = 0.162), cooperation (a large effect: *η*_p_^2^ = 0.261), assertiveness (a medium effect: *η*_p_^2^ = 0.189), empathy (a medium effect: *η*_p_^2^ = 0.259), self-control (a medium effect: *η*_p_^2^ = 0.229), ability to evaluate and convey emotions (a small effect: *η*_p_^2^ = 0.089), ability to utilize one’s positive emotional experience (a small effect: *η*_p_^2^ = 0.088), ability to comprehend and analyze emotions (a small effect: *η*_p_^2^ = 0.080) and ability to control emotions (a small effect: *η*_p_^2^ = 0.079). We did not find studies that have examined the impact of positive behavior skills development programs through sports on 15-16-year-old adolescents attending basketball sports schools. This is one of the arguments for the relevance of this study, aiming to fill this scientific gap. Nevertheless, our study results partially coincide with the findings of other authors who conducted research on this topic. Gülay et al. (2010) conducted a study with 9th-grade (15–16-year-old) adolescent athletes. During the intervention experiment involving 44 adolescents, it was found that a 12-week development program, consisting of 12 activities of 80 min each, was statistically significant for the ability to control emotions (a large effect: *η*_p_^2^ = 0.94) (Gülay et al., 2010). In another intervention experiment involving 103 14–15-year-old rugby players, it was revealed that a 3-month post-school development program (frequency of once a week) was statistically significant for adolescents’ prosocial behavior in sports skills (a medium effect: *η*_p_^2^ = 0.14) (Parise et al., 2015). Another intervention experiment with 11-15-year-olds was conducted by García-García et al. (2020). During the experiment, which involved 57 adolescents, the authors sought to determine the impact of a personal and social skills development program through sports. The authors confirmed the effectiveness of the development program, as a statistically significant effect was found in the skill scales of the participants: taking responsibility (a medium effect: *η*_p_^2^ = 0.197), taking social responsibility (a small effect: *η*_p_^2^ = 0.074), and prosocial behavior (a large effect: *η*_p_^2^ = 0.572). The personal and social skills development program consisted of 29 activities, with each activity lasting 55 min, distributed over 5 months with a frequency of 2 times per week (García-García et al., 2020). Malinauskas and Malinauskiene (2021) investigated the impact of a 12-h (48 activities of 15 min each) social and emotional skills development program through sports on 16-17-year-old adolescents. The intervention experiment revealed a statistically significant effect of the training program on adolescents’ (n = 104) social and emotional skills development through sports in the following skill scales: cooperation (a small effect: *η*_p_^2^ = 0.066), assertiveness (a small effect: *η*_p_^2^ = 0.093), empathy (a small effect: *η*_p_^2^ = 0.076), self-control (a small effect: *η*_p_^2^ = 0.043), ability to evaluate and convey emotions (a small effect: *η*_p_^2^ = 0.039), ability to utilize one’s positive emotional experience (a small effect: *η*_p_^2^ = 0.048), ability to comprehend and analyze emotions (a small effect: *η*p2 = 0.052), and ability to control emotions (a small effect: *η*_p_^2^ = 0.124) (Malinauskas and Malinauskiene, 2021). Meanwhile, Koszałka-Silska et al. (2021b), who investigated the impact of a social skills development program through sports on 15–16-year-old adolescents, revealed that a 12-week program, consisting of 45-min training sessions conducted twice a week, was statistically significant only for the development of assertiveness skills (a small effect: *η*_p_^2^ = 0.07). Another sports-based skills development program was effective in enhancing social skills in 15-year-old adolescents (Akelaitis and Malinauskas, 2016). During the intervention experiment involving 51 adolescents, it was found that a 35-activity (15-min duration each) social skills development program through sports was statistically significant for enhancing adolescents’ cooperation (a small effect: *η*_p_^2^ = 0.09), assertiveness (a medium effect: *η*_p_^2^ = 0.14), and self-control (a small effect: *η*_p_^2^ = 0.01) skills (Akelaitis and Malinauskas, 2016).

We consider that discussion is necessary, how the content, or delivery of the positive behavioral skills development program might need to be modified if it were implemented with athletes involved in individual sports rather than team-based ones like basketball. Thus, if the aim is to develop a positive behavioral skills program for athletes involved in individual sports, content and delivery of the program itself would have to be slightly modified (Bessa et al., 2019). It is likely that the implementation of such a program should not be limited to the specific environment of an individual sport, as the skills developed in this curriculum can only be successfully developed in the context of a social relationship that is common in the context of sporting games (Bessa et al., 2019). The development of these skills would therefore require educational activities that are group/team-based (Bessa et al., 2019), but not exclusively focused on the elements of the basketball game. Consequently, the content of the educational activities would also have to change, as some of the activities in the present study focused on basketball. For example, the cooperation skill educational activity is exclusively focused on the basketball environment, so this activity should be adapted to suit the development of an athlete from individual sport.

There is also a need to discuss who should or could be responsible for implementation of behavioral skills training program in practice. We think that a positive behavioral skills program would be best implemented by any coach working in a sports school, as he or she would not only have the technical knowledge of the sport, but also the ability to develop positive skills such as cooperation, prosocial behavior or ability to control emotions (Newman et al., 2021). As a coach needs to have specialized knowledge of positive psychology principles and pedagogical strategies, he/she is best placed to combine sporting activities with activities to develop positive behavioral skills (Newman et al., 2021).

In summary, it can be stated that the results of our intervention study align with previous research (Fredricks and Eccles, 2006; Mahoney et al., 2005), which indicates that adolescents’ participation in extracurricular or leisure activities (in our case, sports schools) is associated with greater social competence, self-perception, and improved quality of life, facilitating easier formation of various social networks. Thus, integrating positive behavior skills development programs into sports schools allows for the realization of the goals of positive youth development paradigms (Holt et al., 2020; Lerner, 2017).

### 4.1 Contributions and implications

Taking into account the existing scientific gap related to the narrow spectrum of positive behavior skills development programs through sports, based on the results of our study, we propose the creation and implementation of comprehensive positive behavior skills development programs, consisting of positive personal, social, and emotional skills. The comprehensiveness of positive behavior skills is important for realizing the conceptual principles of positive youth development paradigms. The results of this study are significant because the structure and content of positive behavior skills development programs are tailored to 15–16-year-old adolescents. Many educational program studies are focused on other age groups, although as discussed in the literature review, ages 15–16 are crucial for positive behavior skills development. The effect sizes found in the study show that in 7 out of 12 cases (medium or large effect sizes), the positive behavioral skills training program is effective for adolescents aged 15–16 years old attending basketball sports schools. These effects suggest that our approach significantly improves the level of positive behavioral skills among 15–16-year-old adolescents attending basketball sports schools compared to traditional approaches. Such skills improvement through this educational program can lead to better behavioral outcomes, making it worthwhile for coaches to use this approach. Coaches can learn to incorporate social skills training into their coaching practices, helping athletes develop not only physically but also socially and emotionally. The findings of the present study could also be useful for educational programs developers who may consider integrating elements of this educational program into wider education systems. Moreover, findings can lead to the development of training modules that equip coaches with the tools to develop positive behavioral skills effectively. However, it is important to recognize that while the effects in this study are significant, they may vary in different sporting contexts, and this should be considered when applying these findings. Finally, the results of this study are significant because the educational program is oriented not towards physical education classes, which some students are inclined to skip, but towards sports schools, which adolescents choose to attend independently, which may contribute to better educational outcomes.

### 4.2 Limitations and future research

One of the limitations of this study is related to the fact that only male adolescents participating in basketball sports schools were included in the study. This limitation is associated with the data presented in the scientific literature on existing gender differences that lead to different expressions of positive behavior during adolescence. Previous studies (Lipowski et al., 2016) have shown that adolescent girls who participate in sport are more likely than their male counterparts to engage in risk-taking and socially unacceptable behaviors. To address the limitation of our study, future research should prioritize inclusivity by examining both male and female adolescents so that comparative analysis can be made. A study by Hopkins et al. (2022) suggests that mixed-gender studies and mixed-method approaches not only reveal interaction effects but also provide a richer understanding of the dynamics between genders, enriching the applicability of findings. We think it is essential to recognize the differences in emotional and behavioral skills development between the male and female adolescents, to understand their impact on sport and education initiatives, and to encourage future inclusive research. During this study, we also did not conduct a follow-up skill assessment after a period of time, thus failing to reveal whether the skill development program had a long-term impact on adolescents’ expression of positive behavior skills. Therefore, future studies should assess the long-term effects of the intervention through follow-up measurements. Control group of the present study did not receive a social skills training intervention, and this could have several limitations: participants in a passive control group might not feel involved in the study and may withdraw from the study as well as participants may have a preconceived notion that they will not be treated, which may affect their response and bias the results. In future studies, active control group (different intervention) or waiting list control group strategies may increase the rigor of the experimental design and improve the validity of the findings related to the educational intervention. Notably, future research could be carried out in other sports and with a larger number of individuals to make the findings more generalized. In this study, we assumed that the development of positive behavior skills through sports would be effective if implemented not in physical education classes, but in sports schools. In future research, comparative studies could be conducted in which a developed positive behavior skills development program, applied to adolescents of the same age and gender, would be implemented through physical education classes and sports schools, thus determining which sports environment is more suitable for the development of adolescents’ positive behavior skills. Finally, this study did not examine how the acquired skills were applied in other natural settings.

## Data Availability

The raw data supporting the conclusions of this article will be made available by the authors, without undue reservation.
